# Cour D'Appel at Paris

**Published:** 1848-10-01

**Authors:** 


### Medical Jurisprudence in relation to Insanity.

### COUR D'APPEL AT PARIS.

*President, M. le premier President Séguier.* *July* 31.

We have already published some curious details on the subject of *demandes en interdiction*, instituted by the relations of Mademoiselle Scolastique Descharmes. The first application of this kind was rejected in 1842 and 1843. Upon the second, made in 1846, the Court pronounced a judgment which held the matter in abeyance during a year, and appointed M. Debière, notary, provisional committee of the estate of Mademoiselle Descharmes. M. and Madame Daigremont, the latter the niece of Mademoiselle Descharmes, have appealed. M. Delangle, their counsel, addressed the Court as follows:—

About forty years since, a young girl from Lorraine arrived in Paris to seek for a place as servant. After several fruitless attempts, she knocked one day at the door of an apartment in the Rue du Temple, to which she had been addressed.

The door was not opened. An opposite neighbour opened his. The young girl addressed him, saying she was in search of a place. "That is rather strange," replied the neighbour—"I want a servant; will you step in? I had as soon engage you as another." She assented; and Mademoiselle Scolastique Descharmes was at once installed in the service of M. Forestier, brass founder. She continued for many years in this house, where she had to attend the sister of M. Forestier, who died a short time afterwards, aged sixty. Thirty years later, M. Forestier died also, and left her his sole legatee, excepting 10,000 francs which he left to his natural heirs. His fortune was large, and consisted of a house in the Rue Richelieu, producing 10,000 francs per annum, money in the funds, and 60,000 francs capital. Mademoiselle Descharmes doubled the legacies to the family. She retained a very good fortune; about 20,000 francs per annum.

Since she had been in Paris, she had not seen any of her relations. She was rich, but her riches were only a restraint and burden upon her, on account of her industrious and economical habits, which led her to pass her time in cleaning rooms and rubbing furniture. She had become acquainted at M. Forestier's with M. Debière, a notary, who took an interest in her affairs. He proposed to her that he should go to the village of Saint-Nicolas, in Lorraine, her birth-place, and there seek for her relations. This offer having been accepted, M. Debière brought back with him a niece of Mademoiselle Descharmes. She received the child kindly, but in a few days sent her back. At whose instigation will presently be explained. It was the same with Mademoiselle Odile Descharmes, another niece, who was afterwards received with similar good will, and sent away with equal precipitation.

Mademoiselle Descharmes had, when in M. Forestier's service, evinced a strict regard for economy. She cared for nothing but attending to the household duties. She was very fond of her master; let not this point, however, be misunderstood. They were both persons of blameless character. She had had opportunities of marrying, but through fear of displeasing her master she remained single. The result was trying, and was the cause of certain disorders, well known to the medical profession. During more than three years Mademoiselle Descharmes remained always at home. She believed in invisible enemies, whom she designated as *artificial.* She could not, she said, look at the window without seeing those enemies, who made grimaces at her. She had hallucinations; her mind was deranged. Her family called in medical aid. Her state at this time has been well described by M. Trélat, then a physician attached to the Salpétrière—the same who has since become physician of public works—oh, I beg pardon—minister of public works. (Laughter in the Court.)

"I had been informed," says M. Trélat, "that Mademoiselle Descharmes had not left the house for seven years, and that any visitors were disagreeable to her. She received us, notwithstanding, with much politeness. Her relation embraced her, and, at his request, she told us to look at some pictures in her house; she immediately led us herself into her rooms, and called our attention to paintings, engravings, bronzes, and other works of art which were there.

"This visit and the sustained conversation which arose from it made my examination easy, and enabled me to prolong my stay for more than two hours. I was therefore able to speak from time to time to Mademoiselle Descharmes without occasioning her any annoyance.

"Mademoiselle Descharmes replied at first with much ease and propriety, and I could not, during the first hour of my visit, extract a single unreasonable idea from her, with this one exception—I am unable to go out, because the men will not allow me; the men have behaved wickedly to me; I will go out when all this shall be at an end.

"When will this be at an end?—I don't know.

"What do you mean by saying 'I don't know;' what is it that should come to an end?—Ah! I don't know.

"To all questions on this subject, repeated in different ways, she only replied in the same words, 'I don't know,' and yet expressed herself on every other subject with perfect clearness and precision.

"You have, said I, two very beautiful statuettes of Voltaire and Rousseau, have you also their works in your library, and do you read them?—I don't like Voltaire.

"Why?—I think him too dry and satirical.

"And Rousseau?—I think him too weak; he is always tacked to women's petticoats, and allows himself to be deceived by them.

"Do you read much?—Sometimes in the evening.

"How do you generally employ your time?—I keep my rooms clean. I am nothing but a cook—a servant. I rub the floors, I dust the furniture. I am my own servant.

"What food do you prefer?—Beef.

"Do you never eat other kinds of meat, or game?—Seldom; I like beef; I like the rump.

"Mademoiselle Descharmes was pleased at my observations about her health, and appearing to confide in me, she said, 'You are a doctor, I suppose, from your questions?' I answered 'Yes, and therefore I would persuade you to go out. You have not visited our public gardens for a long time; if you were to go to the *Jardin des Plantes*, you would see some new animals; there is a fine collection of monkeys which disport themselves to the great satisfaction of visitors, in an immense cage exposed to the sun, and to every one's gaze.'

"I had accidentally touched a tender chord, and penetrated at once the secret of her disordered state of mind. 'Ah, yes, indeed,' said she, 'the monkeys! They are a fine sight, those monkeys! They did me harm enough to prevent my wishing to see them, when they came continually to make grimaces and insult me, when they pulled my legs, when they split my head open, and abused and mocked me.'

"How could monkeys treat you in this manner and abuse you? You never had monkeys in your house; and, besides, monkeys cannot speak?—I saw them as well as I see you.

"You must have been dreaming—have seen them in your sleep?—Oh, no; I was not asleep.

"In what terms did they abuse you?—They said, and wished to make me do, the most horrible things; and when I refused, they threw me under the bed, made black pudding of me, placed me in a hearse, took me to a cemetery, and then made me eat corpses.

"Have you only had to complain of monkeys?—Monkeys, and men who are no better than they are, and who overwhelm me with nonsense and abuse.

"How do they abuse you?—They call me their little mother!

"Do you consider that very gross abuse?—What! Am I their little mother? Ought they to indulge in such familiarity in addressing me?

"During the conversation, Mademoiselle Descharmes said that she would not be allowed to go out until her subscription to the publications of the Museum at Versailles was completed. These publications, which she really takes in, she preserves in a chimney in one of the rooms. They are, according to her, of a size and weight which could not be lifted by fewer than six men. I have seen this collection in *folio*," says M. Trélat, "which amounts in fact to a large size, but is at least six times smaller than Mademoiselle Descharmes' description of it." M. Trélat having noticed a charming picture in the style of *Duval-Lecamus*, Mademoiselle Descharmes exclaimed, They are good likenesses'—the picture represents a village scene—'They are,' said she, 'family portraits.' She listened to me with attention, and was even touched for an instant when I spoke of the pleasure and amusement which she would derive from returning to ordinary life and habits. I resumed thus:

"Do you look out of the window sometimes, as you do not go out?—Never.

"Why not?—Because it is impossible.

"Your health suffers much from such seclusion, and it is not surprising that you should suffer from complaints which are but a consequence of it, and which you wrongly attribute to other causes. Come to the hospital where I live; you will see there more than 5000 persons treated with humanity, attended and consoled as much as possible in their affliction. Would you like to inspect this grand and useful establishment? see the whole of the cooking performed by seventeen persons; and visit a laundry where 8000 shirts are washed every week? Will you come?—Perhaps I will, some day.

"Why not come now?—That would be impossible.

"You will grant, notwithstanding your modesty, which is remarkable, that you are kind-hearted and generous. These virtues must be limited in their exercise and practice by your complete seclusion. Beneficence should be able to choose proper objects; charity, without sufficient caution, often does harm instead of relieving its object. Money given to relieve distress may be spent at the public-house in drinking. You cannot, as you do not go out, search for and discover unfortunate widows, with families of poor children whom they cannot support?—Here Mdlle. Descharmes interrupted me suddenly, and said, with much volubility, 'Children, children! There are too many of them in the world; why do people have so many children! Men are unreasonable in having such large families of wretched people.'

"These wretches, once existing, ought not to be punished for being born. It is a duty and a virtue to relieve them?—I do give to them."

M. Trélat concludes with the following reflections:—"It is remarkable to what length the conversation was carried before discovering the delusion of Mdlle. Descharmes. The interrogation of a lunatic often requires a great number of subjects to be started, and many details to be entered into, for it is rare that monomaniacs, for example, start themselves the idea which torments them. It must be offered before they will accept it; hence the necessity of a jocose and rather discursive manner of questioning them."

M. Trélat concludes his report with the opinion that Mdlle. Descharmes is insane; that she labours under melancholy or monomania, taking its character from the unreasonable seclusion in which she has remained for seven years. Like all monomaniacs, she mingles with her principal delusion unreasonable ideas relative to the Museum of Versailles, and the monkeys. She has hallucinations of sight and hearing, and delusions.

"Deranged persons," says our authority—Esquirol—"have almost all hallucinations, and when those are not discovered, it is because they dissimulate, and the delusions have not been arrived at. Amongst a hundred lunatics, eighty at least will have delusions."

It is evident that the reason of Mdlle. Descharmes, long unsound, was unable to resist the difficult trial of sudden good fortune: an additional confirmation, if such were required, of the truth of the remark, that there is one thing more difficult to bear than adversity, and that is prosperity.

This remarkable certificate, resumed M. Delangle, has been confirmed by another certificate of Dr. Métivier, who arrived at the same conclusion.

Resting on these facts a *demande en interdiction* was made by her niece, Mdlle. Adèle Descharmes, wife of Daigremont. A meeting of the family was called; it was composed of six relatives or friends. There were three in favour of an *interdiction*, and three against it. A word with regard to the latter. We have seen in the newspapers a man, accused of theft, produce a certificate from his landlord, which declared that his conduct was excellent, because he paid his rent regularly. Well, one of these members of the family council, who was opposed to the *interdiction*, was the proprietor of the house inhabited by Mdlle. Descharmes, and according to him, she could not be mad, because she had continued to occupy her apartments. M. Trouillebat, the judge (too early snatched away from his jurisdiction!) presided over the family council; it was his opinion that Mdlle. Descharmes was afflicted with very serious monomania, and that there were sufficient grounds to justify having recourse to the law regarding insane persons.

A jury proceeded with the examination. There, Mdlle. Descharmes, perceiving the object of the attack directed against her, was on her guard, and replied rationally. At length, on the 29th April, 1842, judgment was pronounced, that, although Mdlle. Descharmes might have shown some eccentricity, nevertheless, the examination showed that she was *compos mentis*, and the application was therefore refused.

On appeal, M. and Mdme. Daigremont produced the conclusive certificates of MM. Trélat and Métivier. A decree of the 25th July, 1842, ordered that M. Demonmerqué, counsel, should proceed to make another examination, and that Mdlle. Descharmes should be examined at intervals by Doctors Andral, Ferrus, and Bleynie.

The report made on the 13th April, 1847, by these physicians, established—1st. That Mdlle Descharmes experiences hallucinations, the cause of which may be traced to a remote period; that she believes in enchanters who place persons in her way, to make grimaces at her, or insult her with indecent propositions. 2nd. That inasmuch as she is not obliged to leave home, she cannot be considered to be an insane person, against whom it is necessary to take precautions. 3rd. That there is room for uneasiness on the score of the extravagance which she might enter into, or the temptation which she might hold out to designing persons; and that it would be proper to give her a *conseil judiciaire.*

I now come to the examination which was made in the presence of M. Demonmerqué, the counsel.

After several circumstantial details on the subject of the management of her property, the Counsel asked her, How do you pass your time?—I work, I sew; I wait even upon my housekeeper (laughter). For my part, I have no regular hours; I eat when I am hungry—eat anything, like a trooper (laughter).

"Have you continued to go out since your visits to the members of the court?—I have not been out since that time.

"When you last went out, did you not go to the *Jardin des Plantes?*—Yes, sir; I went also to Vincennes, and other places.

"Were you pleased with the *Jardin des Plantes?*—No; I dislike slavery, as much for beasts as men.

"Though the animals are all in captivity, some of them are very amusing; the monkeys, for instance?—I dislike monkeys; their grimaces disgust me.

She was then questioned about her relations whom she had seen, and the child whom she had sent back.

"Why did you not keep her with you at least for a few days?—I don't know; I had no reason for not keeping her. I was afraid of ingratitude; moreover, I never was happy.

"You do not, however, appear to have led an unhappy life; you have, from your youth, met with good masters, and, in short, M. Forestier showed his sense of your good behaviour?—Good Heaven, sir, how little you know about the hard work which I had to do in his service, looking after the housekeeping and the work-shop! M. Forestier was a person of rough manners; his voice inspired me with awe; he was attached to me; he wished to make me presents, which I would not accept. I might have been married; M. Forestier knew that I might; he received letters on that subject—he did not oppose it; however, I would not consent.

"What prevented your marrying?—It was a supernatural power; I cannot tell you more.

"What do you mean by a supernatural power?—I know that there is neither sorcery nor witchcraft, and that human beings are guided by reason.

"Who, then, could have prevented your getting married?—I should have liked to have had a husband, a support, some one at home with me; I should have been happy if I could have opened my heart to another; but I was prevented.

"If M. Forestier had asked you to marry him, would you have accepted his hand?—I loved him as a father, but I never could have felt towards him as I should towards a husband.

"You must have been well acquainted with Mdlle. Adêle Descharmes, the wife of Daigremont?—M. Debière found her out in Paris, and introduced her to me; but I have not seen her for a year. They say that she is the cause of my troubles.

"When M. Forestier bought that pretty picture, by M. Duval-Lecamus, what impression did it produce upon you when you first saw it?—M. Forestier called me to look at it, and I thought I recognised a resemblance to my brother and one of my nieces; I do not know my relations; I could not think that this picture contained their portraits. I lost my father when I was very young; he was a master carpenter; he was arrested during the revolution because he would not assist in the destruction of religious monuments—so my relations have told me.

"When you are alone, do you read?—No, sir; I dislike reading, because what people write is different from what they practice, and often the reverse of what they think. I have read a good deal, and it is for that reason that I do not read now.

"But there are books which excite the imagination; *Roland le Ferieux*, and the Enchanter Merlin and his prodigies?—Those are only nonsense—mere stories—and do not interest me.

"You have been well brought up; you have been instructed in the principles of religion; you must wish sometimes to go to church; it would be an inducement for you to go out?—I am religious; I believe in God; I pray to him every night and morning. If I went out, I would go to church.

"Why, then, do you obstinately refuse to go out of doors; you can do whatever you please?—Whatever I please! If I may do whatever I please, I am annoyed to find the law taking note of my actions, which are pure and innocent; and I cannot understand the perseverance with which my conduct, and even my thoughts are scrutinized."

The decision of the Court, on the 26th June, 1843, contrary to the opinion of the counsel whom they had appointed to conduct the examination, was against the *demande en interdiction.*

In 1846, the family, convinced that Mdlle. Descharmes was under surveillance, addressed a complaint, upon which an *information* was granted.

Two reports have been made by the Commissioner of Police for the district of the Mont de Piété, and a person named Gilles, who went with Dr. Leuret to the house of Mdlle. Descharmes, No. 75, Rue du Temple. The doctor put in a certificate, which recapitulates the observations made on this occasion, at the close of the examination of Mdlle. Descharmes.

"She says that she would be glad to go out, but that she dares not do so. From eight years of age, she has been persecuted. The government are in the secret of all this. She likes society; she would have liked to have been married, but every one who presented himself had been prejudiced against her. They would not leave off till they had cut her throat. Those who tormented her were not only a few wicked persons; they were very numerous. At the age of 16, being persecuted just as she is now, she threw herself into the water. Then, and since, she has always been the queen of martyrs. The house which she inhabits is full of *artificial* and invisible people, who say horrible things to her. When any one comes to see her, the artificial people listen to everything which is said, distort everything, and afterwards, when she is left alone, they reproach her with such horrible things, that she dares no longer receive any one. During the recital of her sufferings, she interrupted herself at intervals, to answer the invisible beings, who notwithstanding our presence mocked and insulted her. This conversation proves clearly that Mdlle. Descharmes is afflicted with hallucinations, and is insane. If, as has been observed in others who are in this melancholy condition, she were to arm herself to kill one of the *artificial people* who persecute her, if she were to set fire to the house, in order to burn them all together, she would doubtless be immediately found insane, and therefore, innocent. Nothing can be done with the disorder—it is incurable. A kind and intelligent surveillance should be exercised, to provide against the possibility of mischief arising from her state of mind." (Signed) "LEURET.

"*Paris*, 17 *July*, 1846."

The family having been consulted, upon a majority of four voices to three, it was decided to appoint only a judicial *conseil* for Mdlle. Descharmes. M. Pasquin, the judge, was appointed by the court to proceed with the examination.

She was asked her name and surname?—"Scholastique Descharmes, spinster. Good heavens! how difficult it is to avoid all this writing! M. de Normandie was quite right in saying, it is easier to get money than to keep it! As to my age, I know nothing about it. Put down *more* than the facts; I can't read my register of baptism.

"Are you alone here?—Why should I have any one with me? At the age of nineteen or twenty, I might have been married, but invisible spirits prevented me.

"Are you acquainted with M. de Buce (her lawyer)?—I don't know what you mean. He is a fine protector, indeed! I should die of hunger, if these gentlemen had their way. I am a great fool! I should like trials to be conducted in an open place, in presence of everybody. You came to put a strait-waistcoat on me. Oh, divine justice! can such crimes be permitted on the earth! M. Forestier has been dead ten years; the 2nd of February, this month, spring will begin. Long ago, they used to sing to me, 'Merry month of May, when wilt thou come again?' and they never said anything. M. le President said to me casually, 'This is a pretty affair! they are sharpers!' A voice said, 'Poor innocent! you must get rid of all that. Henry the Fourth, who holds in his hands the sword of peace . . . It is three years since I have lived alone, for the sake of economy—for my tormentors; I go out with a girl, and I am her servant. The greatest sanctity is that of misfortune. It is not sufficient to mould images, he should be a good man. There are two tormentors whom you do not see,—they are placed there by the government, and, as M. Fontaine says, everything mysterious is bad. At night, my tormentors still pursue me; I can't find a corner to go to,—I have dragged my bed all about. It is a mysterious proceeding of the government to enrich us all. It is a new passion of the heart to enrich the government offices. If my fortune had been well managed, I should have 30,000 *livres* per annum. I have 5000 on the *grand-livre*, a house in the Rue Richelieu; I cannot save anything, because there are sharpers. When I see men, I am always afraid of offending them, because it is wrong to give one's hand to any one. Wherever I have been, I have enjoyed everybody's confidence. I have no writings; they will not let me have any pens; from the age of six years they have taken away all my nurses. They put my father in prison; they enlisted my brother before he was old enough, and got him insulted by M. le Curé. I have heard that there are among the nobility some who robbed us. I cannot bear hospitals, schools, and convents,—I like liberty."

The magistrate asserts, that during this examination, Mdlle. Descharmes was very much excited; that she rose and walked about, with the agitation of passion. She first called the magistrate a robber; she exclaimed against the *artificial people*, and designated the magistrate as an *artificial*—adding, that if there were such a thing as justice, they would all be hung.

On this occasion, the unfortunate lunatic was not on her guard, as she was in the presence of M. Trélat and M. Demonmerqué; she completely discovered the sad state of her reason. But notwithstanding all this, a decision of the 5th of May, 1848, considering that there is reason to defer judgment upon the *demande en interdiction*, until the state of her mind can be definitively ascertained, has deferred judgment for a year, and appointed M. Debière administrator of the estate and effects of Mdlle. Descharmes. M. Delangle drew the conclusion, from the latter portions of the documents produced, that an *interdiction* was absolutely necessary. The collateral relations will be accused of cupidity; but they are making use of a sacred right. The course they pursue tends to protect their relation. If anything could augment her malady, it must surely be this continued solitude. Well! let all her wants be attended to, let her be watched with gentle care, and possibly the disorder may yield, partially, at least, to enlightened and assiduous treatment. That was the case with a poor creature who fancied that he was pursued by goblins; his life was a continual struggle with goblins; he had written the history of this combat in several volumes. By dint of care, his family succeeded in reducing to a quiet and slight species of derangement the violent state in which he passed night and day. Now, it must be observed that M. Debière, the notary, has taken possession of Mdlle. Descharmes' apartments, and that he excludes her relations from them so rigorously, that a grating has been put up, in order to intercept them; for you will understand, if the *artificial* people were to present themselves, they must be got rid of. M. Debière has for twenty years managed an income of from 20,000 to 25,000 francs, and M. Debière has had to render no account of his management; during this long period, although Mdlle. Descharmes lives very narrowly, although she almost starves herself, no investment of her savings has been made by M. Debière; and the heir of Mdlle. Descharmes has received from her only 450 francs in all. If, then, the court should not grant the *interdiction*—and that would be a matter for surprise, after hearing the opinion of M. Leuret, chief physician to the Salpétrière, and so good a judge, in such a case, as to the danger attending her present state, and with the examination of Mdlle. Descharmes before us—it will be necessary, at least, to appoint a *conseil judiciaire*, which shall take care of the interests, and also of the person of this unfortunate lady. I am of opinion that, in this case, such a measure becomes an imperative duty upon the court.

M. le premier Président thus said:—"The case will stand over till next Monday, in order to hear M. Paillet, Mdlle. Descharmes' counsel. It will be necessary to ascertain whether, since the information granted upon the complaint and application of the family, M. Debière has been called upon to give in the accounts of which M. Delangle has spoken."—From the *Gazette des Tribunaux*, 1st August, 1848.

